# Revisiting Acute Decompensated Right Ventricle Failure in Pulmonary Arterial Hypertension

**DOI:** 10.2174/0118743064359315250210080743

**Published:** 2025-02-17

**Authors:** Rohit Masih, Vivek Paudyal, Yogendra Mani Basnet, Shaleen Sunesara, Munish Sharma, Salim Surani

**Affiliations:** 1 Department of Hospital Medicine, Hartford Hospital, Connecticut, Hartford, United States; 2 Department of General Practice and Emergency Medicine, Karnali Academy of Health Sciences, Jumla, Nepal; 3 Department of Internal Medicine, Patan Academy of Health Sciences, Lalitpur, Nepal; 4 Department of Population and Public Health Sciences, Keck School of Medicine of the University of Southern California, Los Angeles, California; 5 Department of Medicine, Baylor College of Medicine-Temple Campus, Texas, Tx, United States; 6 Department of of Medicine, Texas A & M University, Texas, Tx, United States

**Keywords:** Pulmonary arterial hypertension, Right ventricle, Right heart failure, Pulmonary Vascular Resistance (PVR), Balloon Atrial Septostomy (BAS), Extracorporeal Membrane Oxygenation (ECMO)

## Abstract

Pulmonary Arterial Hypertension (PAH) is a progressive vascular disease characterized by elevated Pulmonary Vascular Resistance (PVR) leading to Right Ventricular (RV) dysfunction and, ultimately, Right Heart Failure (RHF). Acute decompensation of PAH presents a life-threatening consequence marked by sudden worsening of clinical signs of right heart failure, systemic circulatory insufficiency, and multi-system organ failure. Clinicians are encountering more and more patients with PAH and RHF in the critical care units. These patients require admission and management in a critical care unit until they can be stabilized. The pathogenesis involves an imbalance between RV afterload and its adaptation capacity, ultimately resulting in RV dilation and failure. While the causes of acute decompensation remain subtle in many cases, infections, drug noncompliance, and pulmonary embolism are common culprits. Early identification of signs and symptoms of acute decompensation of RV failure, determination of possible etiology, and timely initiation of optimal treatment approaches are pivotal in avoiding detrimental outcomes. Optimization of pre-load and use of pulmonary vasodilators and inotropic agents are cornerstones of management. In refractory cases, mechanical circulatory support such as Extracorporeal Membrane Oxygenation (ECMO) or Right Ventricular Assist Devices (RVADs) may be necessary. Balloon Atrial Septostomy (BAS) serves as a bridge to definitive therapy, offering decompression of the right atrium and right ventricle. The prognosis of acute decompensated RV failure in PAH patients remains poor, highlighting the critical need for early diagnosis and intervention to improve outcomes. Currently, there are no strict standard guidelines to manage acute decompensated RV failure in PAH patients. We aim to revisit current evidence and practice trends in PAH and its acute decompensation.

## 1 INTRODUCTION

### 1.1 Pulmonary Hypertension

Pulmonary Hypertension (PH) is a spectrum of diseases involving the pulmonary vasculature and is primarily characterized by the mean Pulmonary Arterial Pressure (mPAP) of greater than 20 mmHg at rest [1].

Left-sided heart and lung diseases are the most frequent causes worldwide. It is prevalent across the globe, but approximately 80% of individuals with PH are from developing countries. In such countries, PH is commonly associated with congenital heart diseases, infections like schistosomiasis, HIV, and rheumatic heart disease [2]. While PH has various underlying causes, this article specifically focuses on the pathophysiology and management of acute decompensated Right Ventricular (RV) failure in patients with Pulmonary Arterial Hypertension (PAH).

### 1.2 Pulmonary Arterial Hypertension

Pulmonary Arterial Hypertension is a devastating vascular disease characterized by perivascular inflammation of small pulmonary arteries and progressive remodeling of the pulmonary vasculature, subsequently resulting in RV failure if left untreated [3]. It is a precapillary PH and is hemodynamically characterized by mean Pulmonary Artery Pressure (mPAP) > 20 mmHg, Pulmonary Arterial Wedge Pressure (PAWP) ≤ 15 mmHg, and Pulmonary Vascular Resistance (PVR) > 2 wood units (WU). The details are demonstrated in Fig. (**1**) [4].

### 1.3 Coretips

Pulmonary Arterial Hypertension (PAH) is a progressive and often fatal condition characterized by significant morbidity and mortality. Exacerbation leading to Right Ventricular (RV) decompensation can result in poorer patient outcomes. Effective management of these acute episodes necessitates vigilant monitoring and specialized care in a critical care setting, preferably at centers with expertise in PAH. However, there remains a lack of standardized, evidence-based guidelines for the management of acutely decompensated RV in PAH patients. This review aims to examine current literature and clinical practices, providing insights into evolving approaches to this complex and challenging clinical scenario.

### 1.4 Epidemiology of PAH

In the United States (US), about 10.6 cases per 1 million adults are estimated to be suffering from PAH [6]. The average age at diagnosis is 51.7 +/- 14.5 years. Females are commonly affected, with the female: male ratio being 3.9:1.0 [7]. The causes of PAH are listed in Table **1**.

No cause may be found in about 39% of cases of PAH, whereas approximately 4% are heritable. Connective tissue diseases are the common cause affecting about 15% of patients, followed by congenital heart diseases, portal hypertension, anorexigen-associated PAH, HIV infection, and heritability [8]. Unlike other causes of PAH, patients with methamphetamine-associated PAH (Meth-APAH), which is responsible for drug and toxin-induced PAH, were less likely to be female [9].

If left untreated, PAH typically progresses to right heart failure and death [6]. The estimated median survival age in PAH is 2.8 years. Notably, around 68% of patients do not survive beyond the first year following diagnosis, with survival rates further declining to 48% at three years and 34% at five years [10]. However, recent advances in the medical management of PAH, particularly through therapies targeting key biological pathways, including the nitric oxide-cyclic Guanosine Monophosphate (cGMP) pathway, the prostacyclin pathway, and the endothelin pathway have brought new optimism. The use of combination therapies has contributed to a significant improvement in long-term outcomes, with five-year survival rates rising to 60% in 2015, compared to just 34% in 1991 [6].

### 1.5 Clinical Features of PAH

In the early stages, the patient may be asymptomatic or present with nonspecific symptoms. Features like syncope, angina, and dyspnea may only be present during exertion [11]. PAH is usually detected later in the course of the disease, where most of the cases diagnosed already have severe functional and hemodynamic compromise [8].

The common symptoms of PAH at the time of diagnosis are listed as follows [12]:

- Dyspnea on exertion: It is the most common symptom, and more than 80% of patients seek health care for it.
- Fatigue
- Edema
- Chest pain
- Syncope
- Dizziness/lightheadedness
- Cough
- Dyspnea at rest
- Palpitations.

Most of the clinical features of PAH are because of RV failure during the later course of the disease. These are presented in Fig. (**2**) [4].

The World Health Organization (WHO) has classified PAH into 4 functional classes based on disease severity. The level of risk and estimated 1-year mortality, along with the WHO Functional class (WHO- FC), are presented in Table **2** [4, 13].

### 1.6 Acute Decompensated PAH

Acute decompensated pulmonary hypertension is characterized by sudden worsening of clinical signs of right heart failure with subsequent systemic circulatory insufficiency and multi-system organ failure. It results from an imbalance between the afterload imposed upon the right ventricle and its adaptation capacity. Both systolic and diastolic failure ensues, where the decreased cardiac output suggests systolic dysfunction, and congestive signs suggest diastolic dysfunction of the right ventricle [14]. Worsening dyspnea from baseline, substernal chest pain, worsening leg edema or ascites, abnormal weight gain, new syncope, or pre-syncope could indicate an impending decompensation of PAH and worsening RV failure.

### 1.7 Pathogenesis of Acute Decompensated Pulmonary Hypertension:

Normal pulmonary circulation is a low-resistance and high-compliance system where low pressure is sufficient to pump blood into the lungs for oxygenation. In PAH, there is an increase in PVR, leading to the transformation of the pulmonary circulation into a high-pressure system. As PAH progresses, the RV can undergo adaptation in the form of RV hypertrophy, which can increase the RV contractility up to 4 to 5 folds. The ability of RV hypertrophy is limited. As PVR continues to rise, the RV undergoes dilation in an attempt to maintain adequate circulatory function [15, 16]. The persistent and progressive increase in resistance ultimately results in RV failure as the compensatory mechanisms get overwhelmed and can cause acute decompensated PH. The flow chart of pathogenesis is shown in Fig. (**3**).

The entire discussion about the pathogenesis of acute decompensated PH revolves around PVR. An increase in PVR occurs in PAH because of pulmonary vasculature remodeling. Histopathologic vascular findings appear similar in all forms of PAH and are characterized by vasoconstriction, cellular proliferation, and thrombosis [17, 18]. Pulmonary vascular remodeling involves all 3 layers of the vessel wall, viz. intima, media, and adventitia; intrinsic cells like endothelial cells, smooth muscles, fibroblasts, and inflammatory cells along with platelets. The outcome is progressive, occlusive vasculopathy with endothelial dysfunction leading to impaired production of vasodilators like nitric oxide (NO), prostacyclin, and overproduction of endothelin, further promoting pulmonary vasoconstriction [18]. The progressive remodeling and narrowing of the pulmonary vessels increase the tension on the right ventricular wall. The right ventricle maintains ventriculo-arterial coupling by increasing muscle contractility and wall thickness [16].

According to Laplace’s law [19], the tension on the wall of a sphere is directly proportional to pressure gradient and radius, whereas inversely proportional to its wall thickness.e., T= P*R/2t,

where, T= Tension, P= Pressure gradient, R= Radius and t= wall thickness.

In PAH, the pressure gradient increases because of pulmonary remodeling, which increases the wall tension. The compensatory concentric hypertrophy of the right ventricle thus decreases the wall tension by increasing the wall thickness [14]. These changes occur in the initial stage of PAH, and the patients are either asymptomatic or mildly symptomatic [14] and may remain undiagnosed.

Right ventricular hypertrophy is limited, but PAH is a progressive and persistent clinical entity. Further increase in the afterload of the right ventricle eventually decreases the Cardiac Output (CO). The right ventricle thus undergoes dilatation along with an increase in heart rate in an effort to maintain CO by virtue of Frank-Starling Law [15, 16]. Patients at this stage present with worsening symptoms like dyspnea and syncope [14].

Excessive dilatation of the right ventricle results in end-stage disease characterized by cardiac failure due to ventriculo-arterial uncoupling *via* various mechanisms [16]. Due to the enlargement of the right ventricle, septal curvature becomes convex towards the left and hinders left ventricular filling [14, 20]. Moreover, tricuspid regurgitation can occur, which increases the Right Atrial Pressure (RAP) and further decreases preload to the left heart [14]. On top of it, the increase in right ventricular pressure and mass even impedes blood flow during systole. In normal individuals, the right ventricle is perfused throughout systole and diastole as the aortic pressure is always higher than the right ventricular pressure, irrespective of the cardiac cycle. However, in the case of PH, decreased CO and increased RV pressure impede perfusion to RV during systole and may precipitate RV ischemia [14, 21]. All these changes in the right ventricle impair oxygen delivery and consumption, adversely affect myocardial contractility, alter the synchronous interaction of the left and right heart, and activate the neurohumoral mechanism [15, 22]. Thus, newer therapeutic measures should target the pulmonary vasculature as well as right ventricular remodeling and failure to combat acute decompensation of PH [14].

### 1.8 Possible Contributing Factors for Decompensated PAH

Though acute decompensation is a dreadful complication of PAH, an attempt to find out the cause has been unsuccessful in about 48% of cases. The most common causes found are infection in about 27%, drug noncompliance in 20%, and pulmonary embolism in 3% [23]. The potential triggering factors for acute decompensation of PAH are listed in Table **3** [24-29].

Supraventricular arrhythmias are also responsible for clinical deterioration in patients with PH; however, whether it is a cause or consequence is yet to be answered. Decreased stroke volume because of arrhythmia leads to sudden failure. Restoration to sinus rhythm decreases the mortality, whereas sustained atrial fibrillation carries the risk of mortality as high as 82% within 2 years after the event [14, 30]. Short-term mortality of acute decompensation is very high. The Intensive Care Unit (ICU) mortality itself ranges from 30% to 41% [25, 31], while, 6-month mortality is found to be about 40% [31]. About 2/3^rd^ of the patients dying due to acute decompensated PAH suffer from documented infection [24]. Given its life-threatening outcome, acute decompensation of pulmonary hypertension demands specific management in specialized centers [14].

### 1.9 Diagnosis of Acute Decompensated RV Failure in PAH

Echocardiography and right heart catheterization are essential tools for diagnosing and assessing the severity of RHF in PAH patients. Transthoracic Echocardiography (TTE) can be used non-invasively to estimate RAP by visualizing the Inferior Vena Cava (IVC) diameter. IVC diameter of >2.1 cm and a collapse in diameter of <50% with inspiration is reflective of high RAP [32]. IVC assessment may not be a reliable tool for individuals on mechanical ventilation. A prospective analysis by Jue *et al*. showed a poor correlation between the IVC diameter change and mean RAP [33]. In addition, systolic Pulmonary Artery Pressure (sPAP) can be estimated based on the peak Tricuspid Regurgitation Velocity (TRV) and the TRV-derived Tricuspid Regurgitation Pressure Gradient (TRPG), in the absence of pulmonic stenosis, and considering RAP. Due to the inaccuracies of estimated RAP, the peak TRV is recommended to determine the probability of PH by the 2022 ESC/ERC guidelines for the diagnosis and treatment of pulmonary hypertension [4]. In a multivariate analysis by D’Alto *et al*., TRV of >3.4 m·s−1 independently predicted mPAP ≥25 mmHg and PVR ≥3 WU [34]. Furthermore, the TAPSE/sPAP ratio can be an independent preceptor of Right Ventricular-Arterial (RV-A) uncoupling [35].

Despite the noninvasive modalities, clinicians may still find it challenging to determine an accurate assessment of a patient’s volume status. Additionally, in patients with hemodynamic compromise, it may be necessary to obtain hemodynamic values, including Pulmonary Artery Pressure (PAP), PVR, Cardiac Output (CO), Cardiac Index (CI), and PAWP. In a PAH exacerbation, PVR increases acutely, resulting in increased PAP and subsequent increase in RAP. If not corrected, this leads to progressive right ventricular dysfunction and right heart failure.

Additional testing may be indicated on a case-by-case basis to evaluate potential underlying triggers. An Electrocardiograph (ECG) may be of utility to identify any potential arrhythmias as a trigger. In patients with right ventricular strain, right axis deviation or incomplete or complete right bundle branch block may be noted. An increase in P wave amplitude in lead II can be suggestive of right atrial enlargement. In patients suspected of having a Pulmonary Embolism (PE), a Computed Tomography (CT) angiogram of the chest can be performed to evaluate further. Lab testing should also be conducted to determine potential triggers of acute decompensation, including potential infection.

### 1.10 Management Strategies in Critically ill Patients

These patients require admission to critical care units (intensive care units) until they can be stabilized. The three main aspects of management of acute RHF in PAH involve volume or preload optimization, improved right ventricular contractility, and reduction in right ventricular afterload by means of lowering pulmonary arterial pressure. The use of inotropic agents and pulmonary vasodilators has expanded the therapeutic options available for patients. Pharmacological therapies such as pulmonary vasodilators (prostacyclins, endothelin receptor antagonists, phosphodiesterase-5 inhibitors) are the mainstay of treatment. These medications target different pathways that are involved in the vasodilation or constriction of pulmonary vasculature. In severe cases, inotropic agents, Right Ventricular Assist Device (RVAD), and Extracorporeal Membrane Oxygenation (ECMO) may be utilized as temporizing measures with options such as lung transplantation as destination therapy.

### 1.11 Preload or Volume Optimization

Intravascular volume optimization is one of the most important facets of acutely decompensated PAH. In general, patients with gross volume overload would benefit from diuresis; however, assessment of volume status and overall burden of systemic venous congestion may be challenging. Ultrasonography can be a useful tool to assist with assessing volume status. Hepatic venous flow can also be insightful in estimating volume overload or systemic venous congestion. Flow in the hepatic venous system is normally directed toward the liver, with amplitude typically less than 30%. In the absence of volume overload, the systolic phase amplitude is less than the diastolic phase. An increase in the amplitude of the diastolic phase can be suggestive of mild abnormality, and a reversed systolic phase (away from the liver) is considered severe. Similarly, portal vein Doppler can be used to calculate a pulsatility fraction (PF). Right-sided cardiac volume overload results in hepatic sinusoidal congestion due to pulsatile pressure from the right atrium to the portal vein. The PF can be calculated to determine the degree of pulsatility. PF = 100 [(V_max_ - V_min_) ⁄(V_max_)], in which V_max_ is the maximal flow velocity and V_min_ is the minimal flow velocity. If PF is >50%, it is suggestive of volume overload. In a retrospective study, Denault *et al*. utilized this method and found it helpful in detecting systemic congestion earlier on [36]. In a report of two cases this index was used to help guide and follow up on response to therapy [37]. These modalities, in addition to intra-renal venous Doppler, helped develop the Venous Excess Ultrasound grading System (VExUS). The presence of two severe alterations on Doppler ultrasound with an IVC of ≥ 2 cm has been shown to indicate a high risk of kidney injury in post-cardiac surgery patients [38].

Plasma Brain Natriuretic Peptide (BNP) levels can help predict survival in patients with PAH [39]. In a post hoc analysis of the GRIPHON study [40], Chin *et al*. helped establish the prognostic role of NT-proBNP levels for future events and treatment response [41].

A small-volume challenge can be trialed in hemodynamically unstable patients if Central Venous Pressure (CVP) is not markedly elevated. However, excessive right ventricular preload can lead to further deterioration due to increased right ventricular dilation and less Left Ventricle (LV) filling or ventricular interdependence [16, 42]. More importantly, diuretics should not be held in patients with clear signs of volume overload. Rather, there should be initiation of vasopressor and/or inotropic therapy to support the blood pressure if the patient is hemodynamically unstable. If the patient does develop signs or symptoms of volume overload, it is key to determine whether this is related to the progression of PAH or an adverse effect of PAH-specific treatment. Patients with recent initiation of Endothelin Receptor Antagonists (ERA) may develop peripheral edema with normal Jugular Venous Pulsation (JVP) due to increased vascular permeability related to the therapy [43]. It is important to review not only the medications specific to PAH but also other medications that may contribute to fluid retention (*i.e*., dihydropyridine calcium channel blockers or corticosteroids). Diuretics can be used alone or in combination with other diuretics with various mechanisms of action. An overview of the management is outlined in Fig. (**4**).

### 1.12 Afterload Reduction

The goal of vasoactive medical therapy is to enhance forward flow and augment right ventricular contractility and perfusion. One potential means of achieving this is by decreasing the right ventricular afterload by vasodilation in the pulmonary circulation. There are three main pathways involved in the vascular smooth muscle cells: (a) the endothelin pathway, (b) the nitric oxide pathway, and (c) the prostacyclin pathway.

Endothelin is a substance that acts on receptors located on the vascular smooth muscle cells (*i.e*., endothelin receptors A and B), and by stimulation of these receptors, there is vasoconstriction and proliferation of the pulmonary vasculature. Thus, by inhibition of the endothelin-1 effect on these receptors by Endothelial Receptor Antagonists (ERA) (*i.e*., macitentan, bosentan, ambrisentan), there is less vasoconstriction and an anti-proliferation effect.

In the nitric oxide (NO) pathway, NO directs soluble guanylate cyclase (sGC) to stimulate cyclic Guanosine Monophosphate (cGMP) production, which leads to vasodilation and anti-proliferation. Phosphodiesterase-5 (PDE5) inhibits cGMP; as such, inhibiting PDE5 would increase cGMP and its subsequent effects (*i.e*., vasodilation). sGC can also be activated directly by the administration of nitric oxide (NO) or sGC stimulator (*i.e*., Riociguat). A potential advantage of this is that in areas of the lung where there is inadequate production of NO, these will stimulate the production of cyclic Guanosine Monophosphate (cGMP), resulting in vasodilation. Since the PDE5 inhibitors (*i.e*., sildenafil, tadalafil) increase cGMP by disinhibition, theoretically, this may not be as effective if there is inadequate NO. One potential adverse effect is hypotension. Hence, a combination of PDE5 inhibitors and sGC stimulants should be avoided [44]. Riociguat has been shown to improve the 6-minute walk distance both in patients who were receiving it as monotherapy and in patients on ERAs or prostanoids [45]. In fact, there were also notable improvements in PVR, NT-proBNP levels, and WHO functional class.

Prostacyclin (prostaglandin I2, PGI2), a prostanoid metabolized from endogenous arachidonic acid, is a potent vasodilator. The binding of prostacyclin to the receptor increases intracellular cyclic Adenosine Monophosphate (cAMP), resulting in vasodilation and anti-proliferative effects in the pulmonary vasculature. Prostacyclin derivatives (*i.e*., epoprostenol, treprostinil, iloprost, selexipag) can be administered *via* intravenous, subcutaneous, oral, or inhaled route. Patients who are in acute decompensation should be initiated on a parenteral prostanoid-containing combination regimen. Many of these patients may already be on PAH-specific therapy, and parenteral prostanoids may be added. Early initiation of triple therapy upfront in patients with advanced PAH has shown benefit in terms of functional class [46]. Over longer-term follow-up, it has also been shown to decrease right-sided atrial and right ventricular areas [47].

### 1.13 Inotropic Therapy for RV Failure in PAH

PAH therapies do not directly target the Right Ventricle (RV) [48], and some patients can develop right ventricular dysfunction despite a reduction in PVR [49]. In patients who do develop an acute pulmonary hypertensive crisis with resultant acute RHF, it may be necessary to use inotropic and/or vasopressor support to maintain hemodynamics.

In an analysis by Campo *et al*., the most common cause of hospitalization in PAH patients was acute or acute on chronic RHF, and most of these patients were already on PAH-specific therapy. Despite this, 30.4% of patients required inotropic or vasopressor support. Dopamine was the most commonly used drug and was often used in combination with other vasoactive agents. RHF requiring inotropic support has a very high mortality rate [50]. Ryan *et al*. noted that the choice of preferred inotrope agent in RHF due to PAH varies amongst providers or institutions [51]. The optimal agent may vary based on patient-specific hemodynamics and the desired effects on systemic and pulmonary vascular resistance. Dobutamine increases contractility without much of an impact on pulmonary vascular resistance or tone. In a prospective, controlled animal study, dobutamine decreased pulmonary artery resistance compared to norepinephrine with increased right ventricular contractility and restoration of Right Ventricular-Arterial coupling [52]. Milrinone is an inotropic agent that also decreases systemic and pulmonary vascular resistance. It is a phosphodiesterase 3 inhibitor that affects the myocardium and smooth vascular muscle. It has been shown to be associated with significant increases in right ventricular function, improvement in PVR, pulmonary blood flow, and left ventricular filling in chemically induced pulmonary hypertension [53].

The catecholamines (epinephrine, norepinephrine, dopamine) also have inotropic effects; however, they also increase vascular resistance and may be particularly beneficial in the setting of hypotension to support hemodynamics and help improve RV perfusion. Milrinone is the preferred agent in the event of tachyarrhythmias related to the initiation of inotropes or in patients on beta blockers. The summary of the effects of various inotropes on hemodynamic parameters is shown in Table **4**, with Table **5** showing the mechanisms of action of these inotropes.

A newer class of therapy known as calcium-sensitizing agents has been of interest over the last few decades. One such agent is Levosimendan, which increases the sensitivity of myocardial apparatus to calcium, resulting in increased myocardial contractility. It also has vasodilatory effects and can help decrease coronary vascular resistance and increase coronary blood flow [54]. In a pilot study conducted by Morelli *et al*., Levosimendan demonstrated a reduced right ventricular systolic overload with improved cardiac index and right ventricular ejection fraction in patients with an RV under acute stress [55]. In an animal study, Kerbaul *et al*. saw that Levosimendan restores RV-PA coupling better than dobutamine by decreasing RV afterload [56]. The hemodynamic effects of Levosimendan are evident within 24 hours of initiation, and improved functional status is preserved at 12 weeks [57]. Currently, this drug is not approved in the US; however, it is used in Europe for decompensated congestive heart failure.

### 1.14 Mechanical Circulatory Support

Despite maximal medical therapy, a fraction of patients with PAH may have progressive disease with worsening RV failure requiring the use of Mechanical Circulatory Support (MCS) *via* Extracorporeal Membrane Oxygenation (ECMO) or Right Ventricular Assist Devices (RVADs). Selection of the type of MCS is a multidisciplinary decision based on the patient’s clinical status and the expected duration of MCS as a bridge to definitive therapy. Percutaneous devices and ECMO in PAH are not suitable for long-term use due to the risk of potential device-related complications.

RVADs can help support a failing RV in PAH; however, isolated RVADs may not be efficient in cases of increased afterload as in PAH. Newer devices include dynamic pumps, of which preload and afterload help determine flow. Percutaneous devices allow early intervention without the need for thoracic surgical procedures. The TandemHeart pump and the Impella RP are two such devices that allow flow from the right atrium into the pulmonary artery and directly bypass the right ventricle. This leads to a reduction in right atrial pressure with an increase in pulmonary arterial pressure and left ventricular preload with a subsequent increase in Cardiac Output (CO). The Impella RP is a micro axial-flow catheter requiring one venous access site. The TandemHeart RVAD is an extracorporeal centrifugal-flow pump and two venous cannulas usually deployed *via* bilateral femoral veins. The inflow cannula drains blood from RA into an extracorporeal pump and back to the PA. The Impella RP does not support using an oxygenator; however, with the TandemHeart, one can insert an oxygenator into the circuit to assist with oxygenation. The use of this novel device has been described in patients with RHF secondary to pulmonary hypertension [58]. RVAD increases LV preload, whereas Veno-arterial ECMO (VA-ECMO) increases LV afterload.

Indications for using ECMO include refractory hypoxemia, hemodynamic instability, and failure to wean from mechanical ventilation. VA-ECMO provides cardiac and respiratory support, making it a valuable therapy in Acute RHF. VA-ECMO can provide circulatory support by oxygenating blood and removing carbon dioxide, reducing the workload of the RV. VA-ECMO displaces blood from the right atrium across an oxygenator and delivers it to the arterial circulation. This, in turn, helps improve systemic pressure and left ventricular afterload. Hence, in impaired LV function, a second device may be required to offload the LV [59]. Early initiation and appropriate patient selection are key factors influencing outcomes. Several complications can occur, including bleeding, thrombosis, infection, and hemolysis. The utilization of this therapy may help decrease the time on the waiting list for transplant at the expense of longer intensive care length of stay [60].

### 1.15 Balloon Atrial Septostomy

The creation of a right-to-left atrial shunt (Balloon Atrial Septostomy [BAS]) can be considered as a measure for bridge to definitive therapy. This allows decompression of the right atrium and right ventricle with hopes of alleviating signs and symptoms of RHF. This, in turn, also helps increase left ventricular preload and improve systemic oxygen transport. There have been reports of symptomatic and hemodynamic improvement in the cardiac index after the procedure [61, 62]. In a meta-analysis conducted by Khan *et al*., using BAS leads to beneficial hemodynamic effects, including a reduction in RAP and an increase in cardiac index [63]. This therapy, however, is unlikely to benefit patients with significant RV dysfunction with RHF (RAP ≥20 mm Hg) and/or very high PVR (PVR index ≥4400 dynes sec/cm^5^ per m^2^) or severe hypoxemia and in patients with concomitant LV failure [64].

### 1.16 Lung Transplantation

PAH is a progressive disease, and despite appropriate therapy, at times may be refractory to PAH-specific pharmacotherapy. In such cases, definitive therapy in the form of lung transplantation may be necessary. Patients can undergo bilateral lung transplants or heart-lung transplants, with the latter usually for patients with additional non-correctable cardiac conditions. Ideally, patients should be referred for lung transplantation evaluation if they have worsening functional class requiring escalating therapy or with the use of parenteral targeted PAH therapy [65]. A Lung Allocation Score (LAS) was introduced in 2005 and is calculated using clinical diagnostic factors predictive of survival (*i.e*., PAP, PAWP, CVP, CI, forced vital capacity, functional class, *etc*.) during the following year on the waiting list without a transplant as well as survival during the first year after a transplant. After the introduction of LAS, waiting list mortality has decreased, and there has been an increase in the number of lung transplants [66].

### 1.17 Considerations for Peri-operative Mnagement in PAH Patients at Risk of RV Decompensation

PAH is considered to be a risk factor for perioperative morbidity and mortality, especially in the setting of RHF. It is, therefore, imperative to have multidisciplinary planning and pre-operative risk assessment to have an individualized perioperative plan, as volume shifts, anesthetic medication, and mechanical ventilation can all pose a threat to worsening PH or RV dysfunction. In addition to RV dysfunction, history of Pulmonary Embolism (PE), New York Heart Association (NYHA) functional class ≥II, intermediate or high-risk surgery, and duration of anesthesia >3h have shown to be independent predictors of short-term morbidity. In univariate and multivariate logistic regression analyses, a history of PE, RV hypertrophy, and intraoperative use of vasopressors was associated with postoperative mortality [67]. Intermediate-risk surgeries include carotid endarterectomy and gastrointestinal or abdominal surgery, whereas high-risk surgeries are any major emergent surgery, cardiovascular surgery, or liver transplantation [68]. If perioperative risk is considered to be high, surgery should ideally be carried out at a center with available expertise and resources, including Mechanical Circulatory Support (MCS), if needed in the event of postoperative complications.

Prior to surgery, it is best to lower PVR and improve RV function by means of PAH-specific pharmacotherapy, optimization of intracardiac fillings pressures, and optimization of any cardiopulmonary comorbidities. The optimal goal for hemodynamics is a mean arterial pressure (MAP) > 60-65 mmHg, CI > 2.2 L/min/m2, RAP 5-10 mm Hg, and mean PAP < 35 mm Hg. These values may or may not be achievable in all cases. In any case, during the intraoperative period, systemic hypotension should be avoided to prevent RV ischemia. In addition to this, oxygen should be supplemented to avoid hypoxemia, which would lead to hypoxic pulmonary vasoconstriction and worsen right ventricular afterload.

### 1.18 Special Consideration for Patients Requiring Mechanical Ventilation

Mechanical ventilation in these patients should be aimed at providing adequate oxygenation and ventilation. In addition, caution should be taken to avoid lung overdistension, hypoxemia, hypercarbia, and acidosis as these will lead to worsening of PVR. High PEEP levels (>8-10 cm H_2_O) and larger tidal volumes may cause compression of intra-alveolar capillaries with subsequent increases in PVR. On the contrary, atelectasis should also be avoided, as this can decrease the functional residual capacity of the lung hypoxemia and/or hypercarbia. Therefore, the optimal ventilation strategy would be close monitoring and ongoing assessment to avoid atelectasis and minimize increased alveolar pressure during the intraoperative as well as postoperative period.

### 1.19 Pregnancy and PAH Revisited

PAH increases the risk of complications related to pregnancy, which may be related to physiological changes of pregnancy in the setting of increased PAP with potential RV dysfunction. Risks include fetal hypoxemia, worsening hemodynamics, or volume overload with potential death from RV failure. For pregnant individuals with this comorbid condition, it is important to manage with PAH-specific therapy as outcomes may be worse if left untreated. Medication may need to be adjusted and should ideally be communicated with PH specialists and maternal-fetal medicine providers. ERAs and sGC stimulants (Riociguat) are teratogenic and should be avoided. In such cases, treatment can be continued with parenteral prostanoids.

### 1.20 Prognosis and Outcomes

The prognosis of PAH-related RHF is poor; however, with advances in PAH therapy, survival rates have improved. Early diagnosis and initiation of treatment are crucial for improving outcomes in these patients.

## CONCLUSION

RV failure due to worsening PAH is a fairly common scenario. It significantly impacts the morbidity and mortality of patients with PAH. A comprehensive understanding of the pathophysiology of RV failure in PAH, potential underlying causes, early diagnosis, and initiation of optimal management strategies in a center with expertise in PAH and advanced cardiac critical care facilities are crucial for a favorable outcome. Developing a more concrete guideline-directed management strategy would help achieve a better outcome.

## Figures and Tables

**Fig. (1):**
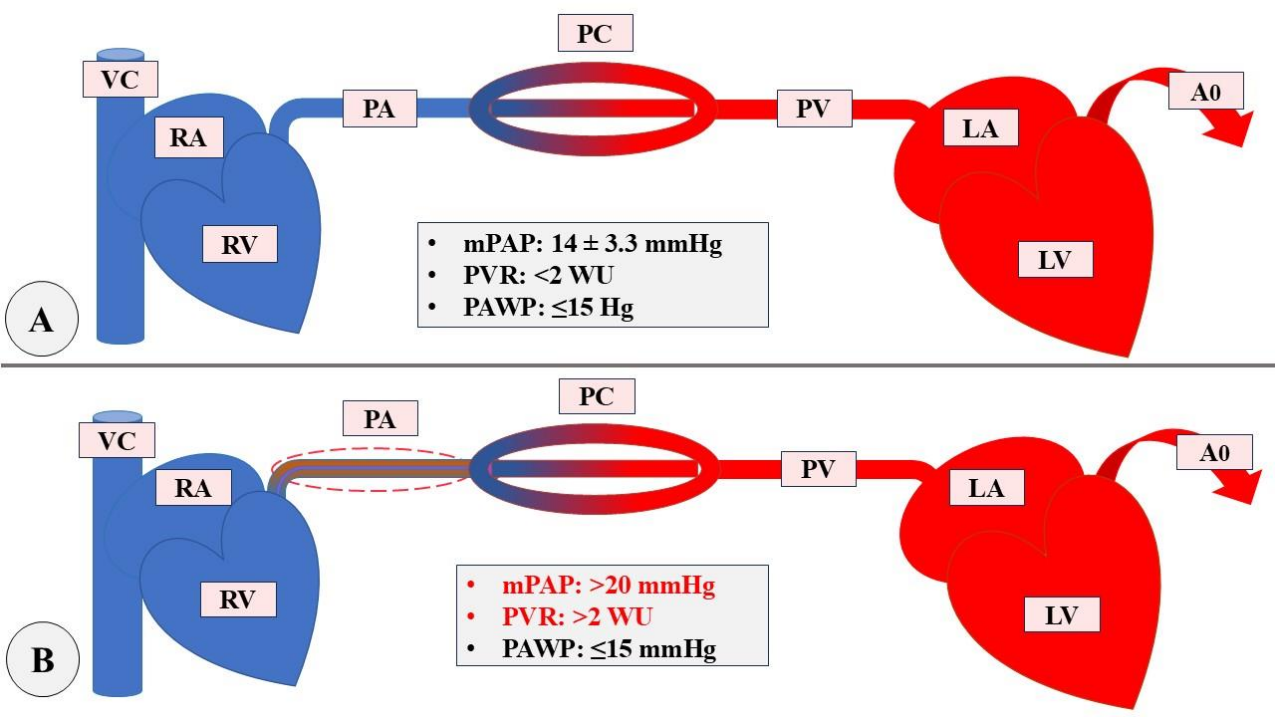
mPAP=(CO×PVR) + PAWP Differences between Normal circulation(A) and Pre-capillary PH(B) [4, 5]. Ao: Aorta, CO: Cardiac Output, LA: Left Atrium, LV: Left Ventricle, mPAP: mean Pulmonary Arterial Pressure, PA: Pulmonary Artery, PAWP: Pulmonary Arterial Wedge Pressure, PC: Pulmonary Capillary, PV: Pulmonary Vein, PVR: Pulmonary Vascular Resistance, RA: Right Atrium, RV: Right Ventricle, VC: Venacava, WU: Woods Unit.

**Fig. (2):**
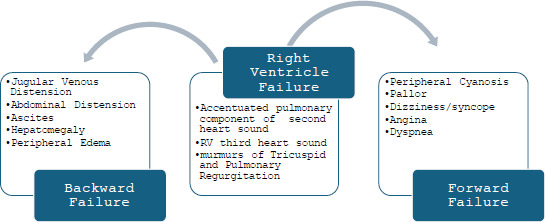
Clinical features of PAH with progression to RV failure.

**Fig. (3):**
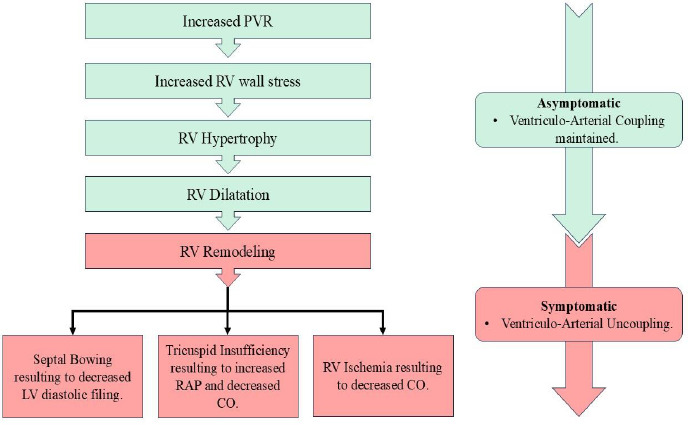
Schematic flowchart depiction of the pathogenesis of Right Ventricular failure in Pulmonary Hypertension. CO: Cardiac Output, LV: Left Ventricle, PVR: Pulmonary Vascular Resistance, RAP: Right Atrial Pressure, RV: Right Ventricle.

**Fig. (4):**
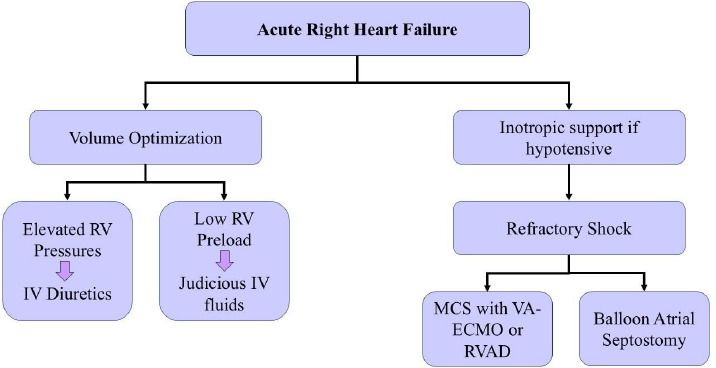
Schematic flowchart of management of Acute Right Heart Failure. ECMO: Extracorporeal membrane oxygenation, IV: intravenous, MCS: Mechanical circulatory support, RV: Right Ventricle, VA-ECMO: Venoarterial extracorporeal membrane oxygenation.

**Table 1:** The causes of PAH.

• Idiopathic PAH • Heritable PAH (*e.g*., Bone Morphogenetic Protein Receptor 2, BMPR2 gene) • Drug- and toxin-induced PAH. o Definite: ▪ Aminorex ▪ Fenfluramine ▪ Dexfenfluramine ▪ Benfluorex ▪ Methamphetamines ▪ Dasatinib ▪ Toxic rapeseed oil o Possible: ▪ Cocaine ▪ Phenylpropanolamine ▪ L-tryptophan ▪ St John’s wort ▪ Amphetamines ▪ Interferon-α and -β ▪ Alkylating agents ▪ Bosutinib ▪ Direct-acting antiviral agents against hepatitis C virus ▪ Leflunomide ▪ Indirubin (Chinese herb Qing-Dai) • PAH associated with: o Connective tissue disease o HIV infection o Portal hypertension o Congenital heart disease o Schistosomiasis • PAH long-term responders to calcium channel blockers • PAH with overt features of venous/capillaries involvement • Persistent PH of the newborn syndrome

**Table 2:** WHO functional class and corresponding level of risk and estimated 1-year mortality risk in cases of PAH.

**WHO-FC**	**Performance Capacity**	**Level of risk**	**Estimated 1-year mortality risk**
**I**	No limitations to physical activity	Low risk	<5%
**II**	Slight limitations of physical activity Comfortable at rest Ordinary activities can cause undue dyspnea or fatigue, chest pain, or near syncope	Low risk	<5%
**III**	Marked limitations of physical activity Comfortable at rest Less than ordinary activities can cause undue dyspnea or fatigue, chest pain, or near syncope	Intermediate risk	5-20%
**IV**	Inability to carry out any physical activity without symptoms of Dyspnea or fatigue, even at rest	High risk	>20%

**Table 3:** Potential triggering factors for acute decompensation of PAH.

**Triggering factors for Acute Decompensation of PAH**
**Infections** **Sepsis** **Pneumonia** **Infective Endocarditis** **Cerebral Abscess** **HIV** **Drug Non-compliance** **Interruption of Prostanoid infusion** **Unplanned modification/withdrawal of Pulmonary vasodilator therapy** **Unplanned modification/withdrawal of diuretics** **Noncompliance of oral drugs** **Pulmonary Thromboembolism** **Cardiac Arrhythmia** **Thyroid Dysfunction** **Hypoxia** **Ischemia** **Trauma/Surgery** **Hypo/Hypervolemia** **Anemia** **Pregnancy**

**Table 4:** Effects of various inotropes on hemodynamic parameters. PVR: Peripheral Vascular Resistance, SVR: Systemic Vascular Resistance.

	Cardiac Output	PVR	SVR
Dobutamine	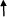	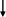	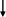
Milrinone	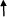	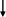	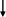
Dopamine	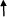	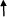	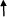
Norepinephrine	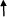	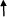	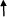
Epinephrine	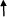	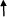 / 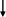	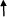

**Table 5:** Mechanism of action of various inotropes.

	Beta 1	Dopaminergic Receptors	Alpha 1	PDE
Dobutamine	+			
Milrinone				-
Dopamine	+	+	+	
Norepinephrine	+		+	
Epinephrine	+		+	

## LIST OF ABBREVIATIONS

ECMO: = Extracorporeal Membrane Oxygenation
Rvads: = Right Ventricular Assist Devices
RV: = Right Ventricular
RHF: = Right Heart Failure
PVR: = Pulmonary Vascular Resistance
PAH: = Pulmonary Arterial Hypertension
PAWP: = Pulmonary Arterial Wedge Pressure
mPAP: = mean Pulmonary Arterial Pressure
Meth-APAH: = methamphetamine-associated PAH
TRV: = Tricuspid Regurgitation Velocity
sPAP: = systolic Pulmonary Artery Pressure
ECG: = Electrocardiograph
CT: = Computed Tomography
RVAD: = Right Ventricular Assist Device
ECMO: = Extracorporeal Membrane Oxygenation
sGC: = soluble guanylate cyclase
cGMP: = cyclic Guanosine Monophosphate
BAS: = Balloon Atrial Septostomy
